# Inhibition of glycogen synthase kinase-3-beta (GSK3β) blocks nucleocapsid phosphorylation and SARS-CoV-2 replication

**DOI:** 10.1186/s43556-022-00111-1

**Published:** 2022-12-12

**Authors:** Tirosh Shapira, Selvarani Vimalanathan, Celine Rens, Virginia Pichler, Sandra Peña-Díaz, Grace Jordana, William Rees, Dirk F. H. Winkler, Iqbal Sarai, Theodore Steiner, François Jean, Steven Pelech, Yossef Av-Gay

**Affiliations:** 1 Division of Infectious Disease, Department of Medicine, Life Sciences Institute, University of British Columbia, 2350 Health Sciences Mall, Vancouver, BC V6T 1Z3 Canada; 2 Department of Microbiology and Immunology, Life Sciences Institute, University of British Columbia, 2350 Health Sciences Mall, Vancouver, BC V6T 1Z3 Canada; 3 Kinexus Bioinformatics Corporation, Suite 1 – 8755 Ash Street, Vancouver, BC V6P 6T3 Canada

**Keywords:** GSK3β, SARS-CoV-2, Host-directed therapy, Host-pathogen interactions, Antivirals

## Abstract

GSK3β has been proposed to have an essential role in *Coronaviridae* infections. Screening of a targeted library of GSK3β inhibitors against both SARS-CoV-2 and HCoV-229E to identify broad-spectrum anti-*Coronaviridae* inhibitors resulted in the identification of a high proportion of active compounds with low toxicity to host cells. A selected lead compound, T-1686568, showed low micromolar, dose-dependent activity against SARS-CoV-2 and HCoV-229E. T-1686568 showed efficacy in viral-infected cultured cells and primary 2D organoids. T-1686568 also inhibited SARS-CoV-2 variants of concern Delta and Omicron. Importantly, while inhibition by T-1686568 resulted in the overall reduction of viral load and protein translation, GSK3β inhibition resulted in cellular accumulation of the nucleocapsid protein relative to the spike protein. Following identification of potential phosphorylation sites of *Coronaviridae* nucleocapsid, protein kinase substrate profiling assays combined with Western blotting analysis of nine host kinases showed that the SARS-CoV-2 nucleocapsid could be phosphorylated by GSK3β and PKCa. GSK3β phosphorylated SARS-CoV-2 nucleocapsid on the S180/S184, S190/S194 and T198 phospho-sites, following previous priming in the adjacent S188, T198 and S206, respectively. Such inhibition presents a compelling target for broad-spectrum anti-*Coronaviridae* compound development, and underlies the mechanism of action of GSK3β host-directed therapy against this class of obligate intracellular pathogens.

**Supplementary Information:**

The online version contains supplementary material available at 10.1186/s43556-022-00111-1.

## Introduction

Coronavirus disease 2019 (COVID-19) is caused by the novel coronavirus SARS-CoV-2, which has infected over 570 million people worldwide, been linked to over 6.4 million deaths, and has precipitated significant social and economic global disruptions [1, 2]. Due to urgency and the limited knowledge of SARS-CoV-2 pathogenesis, development of novel therapeutics has focused on either the repurposing existing antivirals or finding new drugs that target viral entry or replication. Most infected patients clear SARS-CoV-2 infection without treatment through innate and adaptive immune responses, demonstrating that human hosts have a built-in capacity to neutralize these acute infections [3]. At the molecular level, response to infection is mediated by various cellular signaling pathways that activate protective processes including autophagy, apoptosis or even necrosis of infected cells. Consequently, we investigated the modulation of host signaling proteins involved in infection clearance and potentially important for viral replication.

The nature of host function exploitation during viral infection presents an alternate opportunity for therapeutic intervention. Host-directed therapies (HDTs) target host pathways and processes, thereby indirectly restricting viral pathogenesis. Targeting the cell itself may also act as more effective broad-spectrum treatments against various viral strains and alleviate the pressures leading to downstream drug resistance through viral mutations and selection. Previous studies have identified host proteins involved during coronavirus infections as targets of already approved drugs [4, 5], making them eligible potential candidates for use in HDTs. Library screening of FDA-approved drugs for coronavirus replication inhibitors identified Abelson (Abl) kinase inhibitors, including imatinib, as effective against both the 2003 SARS-CoV strain and MERS-CoV in vitro [5]. Moreover, inhibition of another signaling protein, glycogen synthase kinase-3 (GSK3), was found to reduce viral nucleocapsid (N protein) phosphorylation in SARS-CoV-infected VeroE6 cells and overall decreased viral titer and cytopathic effects. Reproduced in the coronavirus neurotropic strain of mouse hepatitis virus, these results indicate that GSK3 is critical for coronavirus N protein phosphorylation and indicates that it plays a role in regulating the viral life cycle [6]. Furthermore, SARS-CoV-2 N protein phosphorylation was recently shown to be absent in GSK3β knock-down cells [7]. As such, GSK3β could serve as a candidate target for COVID-19 host-directed antiviral therapy [8].

The SARS-CoV-2 N protein is an abundant RNA-binding protein critical for viral genome packaging, yet the molecular processes and characteristics of this function are not fully understood. It acts as a phosphoprotein that associates with the viral RNA genome to form the ribonucleoprotein core [9]. Composed of three dynamic, disordered regions that house putative transiently-helical binding motifs, it adopts a conformation of two folded domains that interact minimally such that it remains a flexible and multivalent RNA-binding protein.

GSK3 is a key control kinase of glycogen synthesis [10] and adopts GSK3α and GSK3β isoforms [11], each with differential regulation [12] and tissue expression [13]. GSK3 serves a central signaling role [14] in many regulatory processes through intersection with the PI3K, mTOR, PKB/AKT, WNT, and MAPK pathways [15]. As such, GSK3 is currently a target for drug discovery efforts in Alzheimer’s disease, cancer, diabetes, multiple sclerosis, and others (reviewed [16]). GSK3β has additionally been shown to play a role in bacterial [17] and viral infection control [7]. The efficacy of GSK3 inhibitors against COVID-19 have been observed in existing therapies, including the well-tolerated Enzastaurin and the bipolar disorder treatment, lithium chloride, however clinical therapeutic ranges and in vitro results have not yet been correlated [7]. Despite the confirmed role of GSK3 in N protein phosphorylation during viral replication, variable inhibition results across conditions highlight the need for further screening to identify more selective and potent compounds against this host-derived therapeutic target.

In this study, we screened a GSK3β-focused library of inhibitors in a cellular infection model and identified several potent candidates active against human coronavirus HCoV-229E and SARS-CoV-2. The lead inhibitor, T-1686568, was found to be active against SARS-CoV-2, its variants, and in clinically-relevant immortalized cell line models and human organoids at 1000-100,000 lower concentrations than the previously discussed GSK3 inhibitors.

## Results

### Screening of the GSK3β-focused library

**Fig. 1:**
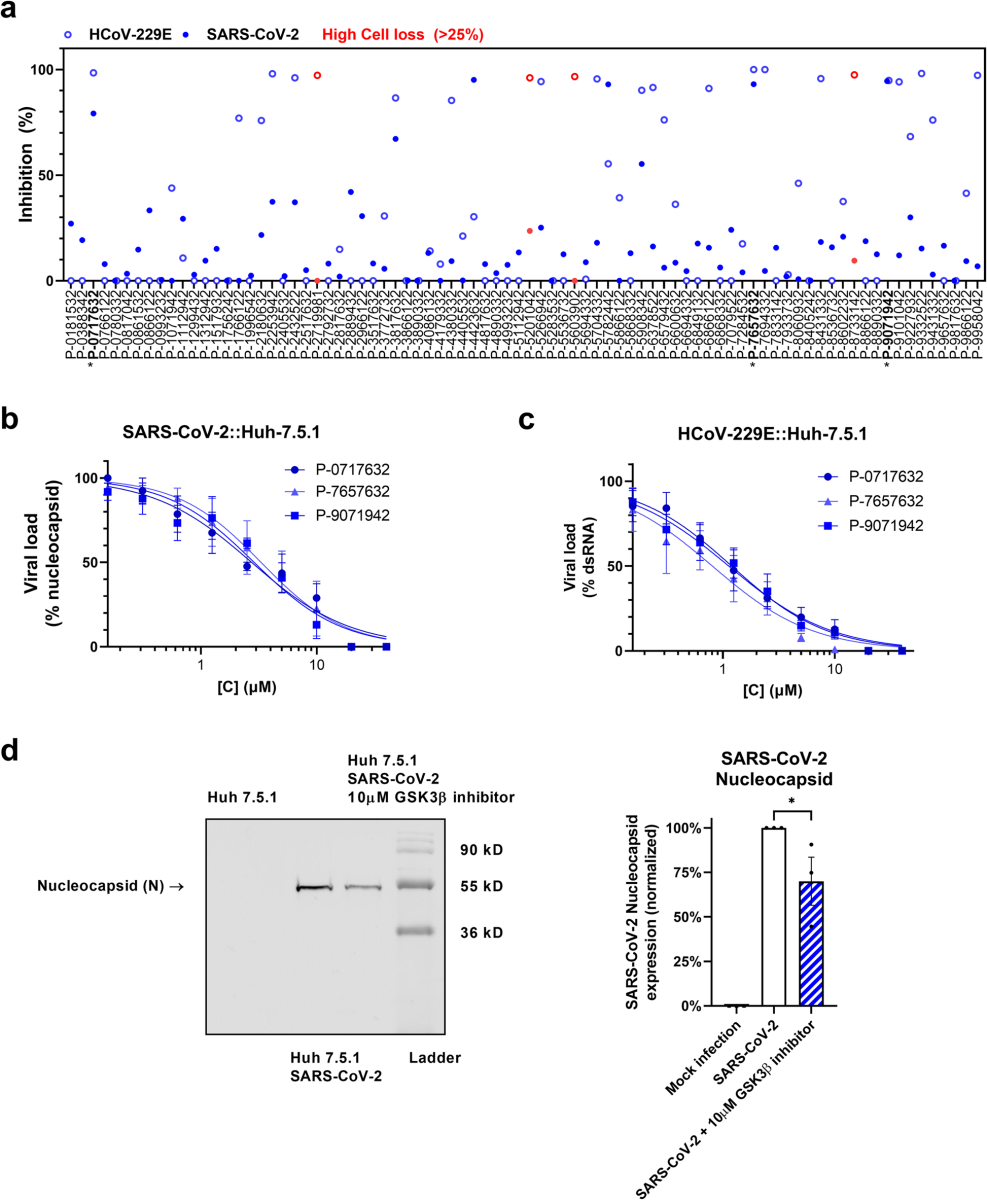
Screening of GSK3β inhibitors. **a** High-content screen of SARS-CoV-2 (full circles) and HCoV-229E (empty circles) infected Huh-7.5.1 cells with the Takeda GSK3β-focused library at a single dose of 10 μM. Inhibition was interpolated to both a non-infected control and an infected, untreated control, quantifying intracellular dsRNA. Cell loss was assessed to avoid quantifying small populations and compounds resulting in > 25% cell loss are marked in red. Compounds marked with an asterisk were further pursued. The Z’ of two independent screens were 0.54 and 0.74 for SARS-CoV-2 and 0.68 for HCoV-229E. **b-c** Dose-response validation of compounds of interest. Each dose is an average of four independent experiments, error bars show SEM. Viral load of SARS-CoV-2 (**b**) was determined by measuring intracellular N protein and viral load of HCoV-229E (**c**) by measuring dsRNA. **d** Western analysis of SARS-CoV-2 N protein, representative blot of four independent experiments

A focused GSK3β inhibitor library [18] provided by Takeda Pharmaceutical Company (Japan) was screened against both SARS-CoV-2 and human alpha coronavirus HCoV-229E-infected Huh-7.5.1 cells and monitored for either dsRNA or N protein levels as markers of infection (Supplementary Table 1). As seen in Fig. 1, a high proportion of compounds active against GSK3β was effective at 10 μM at reducing SARS-CoV-2 and HCoV-229E infection. In measuring N protein, a marker of viral translation, of the 83 screened compounds, 7 compounds (8%) showed ≥50% inhibition, with nearly half (*n* = 40; 48%) of the compounds having some inhibitory effect (> 10%) (Fig. 1a). Screening for inhibition of N protein and dsRNA yielded similar readouts (Supplementary Fig. 1), indicating that relative N protein translation abundance may be used as a reliable proxy for infection and viral load. Probing of dsRNA is an oft-used method, as it serves as a specific marker of viral replication in RNA viruses. Using the dsRNA marker (Fig. 1a), screening against HCoV-229E and SARS-CoV-2 demonstrated that targeting GSK3β resulted in effective viral control in 31% and 19% of the compounds (≥ 50% infection inhibition), respectively. Together, the high ratio of potential hits and relatively low toxicity of the library as a whole support the viability of GSK3β as a target for HDT in COVID-19 and other human coronaviruses infections.

### Dose- and cell-dependent analysis of hit compounds

**Fig. 2:**
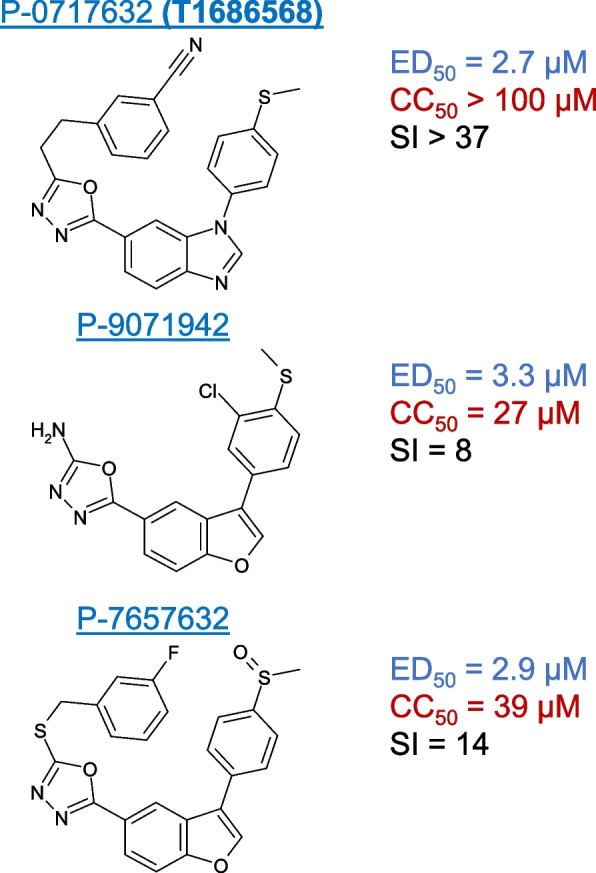
Compound structure, activity against SARS-CoV-2 (ED_50_) and toxicity to Huh-7.5.1 cells (CC_50_). Data is derived from four independent experiments

Three active compounds, T-1686568 (P-0717632), P-7657632, and P-9071942, chosen for their high activity against both viruses, were tested for their efficacy in reducing SARS-CoV-2 and HCoV-229E infection. Dose-response assays determined an effective dose of ~ 3 μM in 50% of cells (ED_50_), with no significant differences observed between the selected inhibitors (Fi. 1b, c). Pre-treatment of cells with the three compounds did not appear to improved antiviral activity (Supplementary Fig. 2). Selectivity Index (SI) was additionally measured, as a value of cytotoxic concentration (CC_50_) over ED_50._ In Huh-7.5.1 cells infected with SARS-CoV-2, T-1686568 was the most tolerated at CC_50_ > 100 μM (SI > 36) while P-7657632 and P-9071942 CC_50_ values were 39 μM (SI =14) and 27 μM (SI = 8), respectively (Fig. 2). T-1686568’s favourable SI value made it a suitable candidate for further analysis; T-1686568 was shown to inhibit GSK3β enzymatic activity with an IC50 of 102 nM (Supplementary Fig. 3) and to reduce N protein levels cells infected with SARS-CoV-2 (Fig. 1d). T-1686568 acts synergistically with the FDA-approved antiviral Remdesivir in HCoV-229E-infected cells. Using the Chou-Talalay method [19], an average Combination Index (CI) value of 0.7 was determined (Supplementary Fig. 4).

### T-1686568 is a potent inhibitor of SARS-CoV2 in various human-derived cell lines and primary organoids

**Fig. 3:**
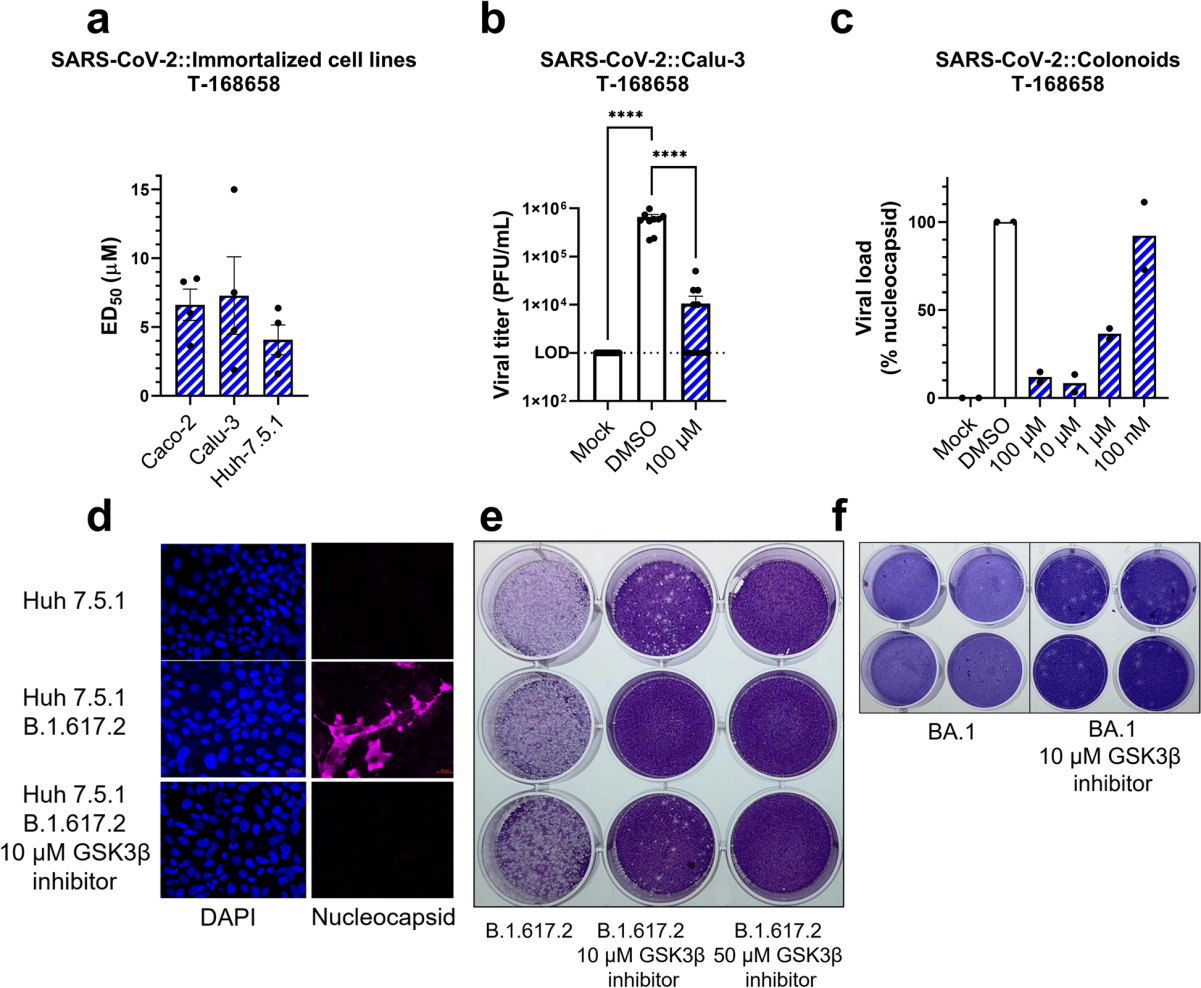
Characterization of T-168658 inhibitory activity. **a** Dose-response inhibition effect of T-1686568 in three infected, immortalized cell lines. Data is averaged from four independent experiments; error bars are the SEM. **b** Viral titer from 48-h infected Calu-3 cells, with (100 μM, no host-cell toxicity observed) and without (DMSO) T-1686568 from 12 independent infections; error bars are the SEM. One-way ANOVA with Bonferroni multiple comparison correction, *P* < 0.0001. LOD – Limit of Detection. **c** Semi-dose-response inhibition effect of T-1686568 in primary donor-derived colon organoids, 72 hrs post-infection. Viral load interpolated to both non-infected cells (mock) control and an infected, untreated control, quantifying intracellular N protein. GSK3β inhibitor activity against the SARS-CoV-2 variants B.1.617.2 (delta) and BA1 (omicron). Immunofluorescence confocal images (**d**) of Huh-7.5.1 infected with B.1.617.2 with or without 10 μM T-1686568 treatment for 48 h. Host nuclei (blue), nucleocapsid (magenta), are shown, with scale bar (red) = 20 μm. **e** Plaque assays of B.1.617.2-infected Vero E6 cells, 2 days post-infection. **f** Plaque assays of BA.1-infected Vero E6 cells, 2 days post-infection

Relative inhibitor activity is often influenced by variability in different cell infection models [20]. While liver-derived Huh-7.5.1 cells were suitable for screening, initial validation, and toxicity studies, we evaluated T-1686568 potency in two other common SARS-CoV-2 infection models, colon Caco-2 cells and the clinically-relevant lung Calu-3 cells. In all investigated cell lines, T-1686568 provided a similar robust activity, with the ED_50_ ranging 4 to 7 μM (Fig. 3a). This observation indicates that GSK3β phosphotransferase activity is needed for SARS-CoV-2 infection, independent of host cell type. While these results show a reduction of intracellular viral markers, they may not directly translate to a reduction in viral particle production and release. To evaluate whether T-1686568 equally impacted infective particle release from SARS-CoV-2 infected Calu-3 cells and the inhibitory effect at ED_99_, viral titres were measured and showed a significant 2-log reduction 48 h after infection and treatment (Fig. 3b).

To approach a patient model for SARS-CoV-2, donor-derived organoids or induced pluripotent stem cell-derived organoids are used as the closest analogs. These organoids can be infected with SARS-CoV-2, have greater cell variability than monoclonal immortalized cell lines, and do not carry tumorigenic artefacts [21, 22]. Using donor intestinal organoids monolayer infected with SARS-CoV-2, T-1686568 also showed a proxy efficacy in a dose-dependent manner to greatly decrease intracellular N protein levels (Fig. 3c). In addition to the original SARS-CoV-2 strain, T-1686568 reduced intracellular viral titres in the B.1.617.2 and BA.1 variants (Fig. 3d-f).

### T-1686568 treatment results in population reduction in viral markers, and in accumulation of viral N protein in remaining infected cells

**Fig. 4:**
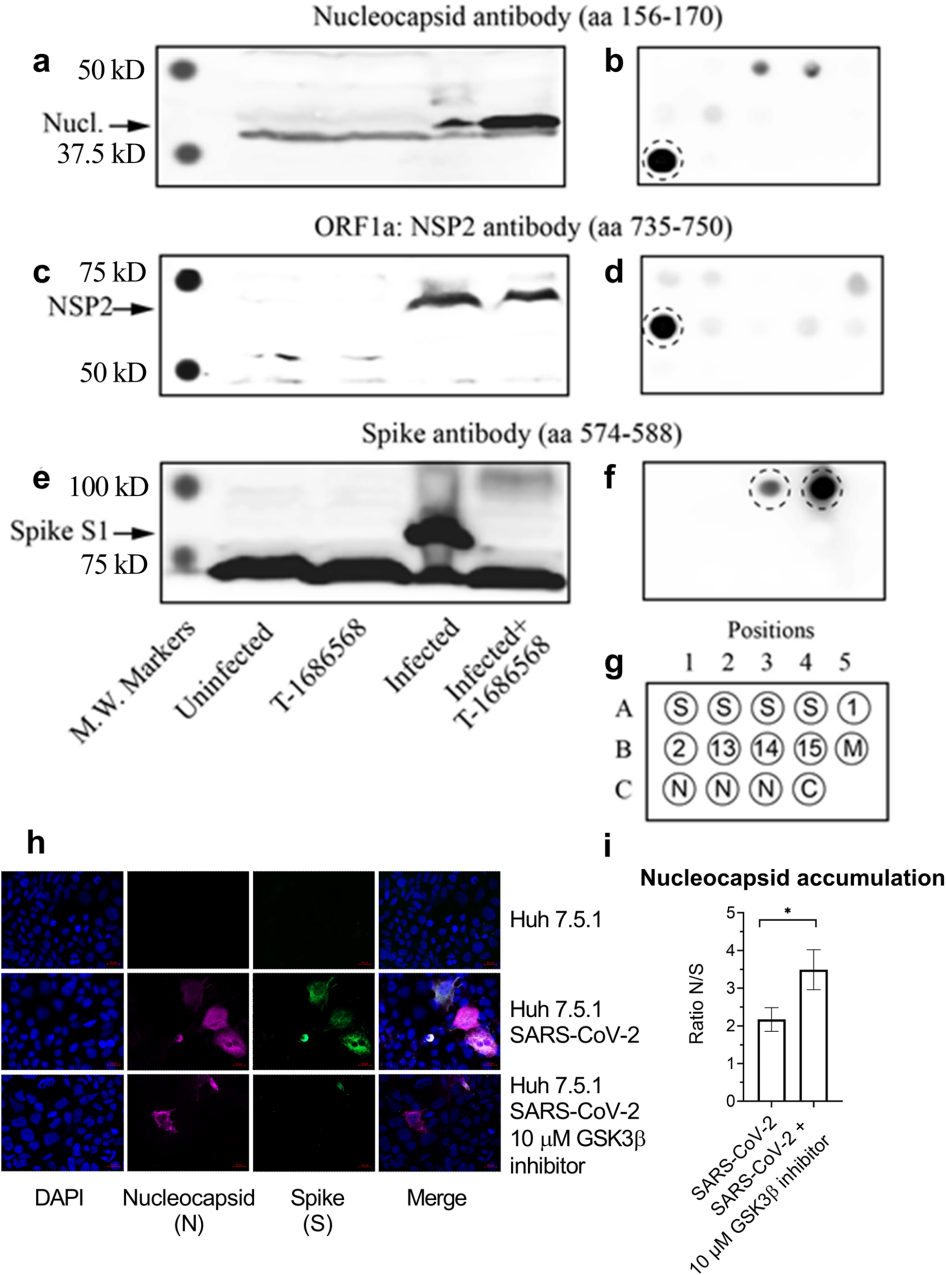
Presence of SARS-CoV-2 proteins in infected Huh-7.5.1 cells treated with and without T-1686568. **a, c, e** Cells were pre-treated with 10 μM T-1686568 for 3 hours and then incubated with the virus for 2 days prior to harvesting and western blotting. **b, d, f** Recombinant preparations of SARS-CoV-2 protein and peptides were robotically spotted on to nitrocellulose membranes as configured in (**g**) and then immunoblotted with the following antibodies that were generated against peptides patterned after sequences in: **a, b** the nucleocapsid (Nucl.; NNCOV2N-1 raised against amino acids 156-170); **c,d** the non-structural protein NSP2 in the ORF1; NNCOV2-1A-2 raised against amino acids 735-750); and the spike protein S1 subunit (Spk S1; NNCOV2S-10 raised against amino acids 574-588). Locations of expected positions of target proteins and peptides are circled in the dot blots shown in Supplementary Fig. 7. Examples shown are representative of triplicate experiments. **h** Confocal images of Huh-7.5.1 infected with SARS-CoV-2 with or without 10 μM T-1686568 treatment for 48 h. Host nuclei (blue), nucleocapsid (magenta), spike (green) are shown, with scale bar (white) = 20 μm. **i** Quantification of fluorescent nucleocapsid and spike proteins is averaged from 1512 total cells, minimum 397 cells/condition from three independent experiments, Student t-test * *p* < 0.05

The Spike (S) protein of SARS-CoV-2 is another important outcome of viral translation, and one that is not known to be phosphorylated directly by GSK3, so can be used as a reference for viral load. S protein expression in infected Huh-7.5.1 cells was reduced by treatment with ED_80_ T-1686568 (10 μM), yet the non-structural protein (NSP2) and N protein could still be detected, with an apparent accumulation of the N protein (Fig. 4). The specificity of the antibodies used to monitor the levels of the S, NSP2 and N proteins was validated with dot blots with recombinant versions of these and other SARS-CoV-2 proteins and peptides (Fig. 4). Fluorescence microscopy confirmed overall population reduction in N protein and S protein levels were reduced upon treatment with ED_80_ T-1686568 (Fig. 4h), yet importantly N protein was accumulated in remaining infected cells (Fig. 4I). We also observed reduction of cellular syncytia formation and filopodial protrusions associated with T-1686568 treatment (Supplementary Fig. 5).

### GSK3β phosphorylates primed S180/S184, S190/S194 and T198/S202 sites of the SARS-CoV-2 N protein

Bioinformatic in silico and experimental data from multiple mass spectrometry studies identified 37 phosphorylation sites on the coronavirus N protein (Supplementary Fig. 6). At least 15 of these confirmed phosphorylation sites were located at Ser-176 to Ser-276 within the arginine-serine-rich (RS) domain, with many complementing consensus phosphosite recognition sequences in GSK3 [20]. Using in vitro kinase assays, nine different recombinant host kinases were used to phosphorylate a set of SARS-CoV-2 phosphopeptides, synthetic nucleocapsid peptides modeled after eight phosphosites in the RS domain. Half of these peptides were artificially pre-phosphorylated at sites that were predicted to be subsequently secondarily phosphorylated by GSK3. GSK3β further phosphorylated all four of these phosphopeptides and protein kinase C-alpha (PKCα) was observed to be able to phosphorylate three of the four pairs of peptides provided that they were not already pre-phosphorylated at the GSK3 priming phosphosites (Table 1). These in vitro assays showed that GSK3β effectively phosphorylates peptides containing S180/S184, S190/S194 and T198/S202, which have been previously primed (i.e., phosphorylated) in the flanking S188, T198 and S206 phosphosites, respectively. Furthermore, GSK3β and PKCα were able to phosphorylate the full length nucleocapsid protein (Table 2). GSK3β and PKCα both phosphorylated the peptides to varying degrees, but unexpectedly the combination of the two did not yield overall higher phosphorylation activity. This is likely due to incomplete priming of the peptides by PKCα in the limited duration of the phosphotransferase assays (Table 2).

**Table 1:** In vitro phosphorylation of synthetic peptides modeled after SARS-CoV-2 nucleocapsid protein phosphorylation sites by protein kinases using the ADP-Glo method

Peptide Name	Peptide Sequence	CDK2 + Cyclin A2	CK1a1	CK1d	CK1g1	CK2a1	ERK1	GSK3b	PKACa	PKCa
SCV2-N [177-190] - pS188	RGG**S**QAS**S**RSS(**pS**)RS	NT	NT	NT	NT	NT	NT	1209	128	389
SCV2-N [177-190]	RGG**S**QAS**S**RSS**S**RS	NT	NT	NT	NT	NT	NT	107	162	1320
SCV2-N [180-193] - pS190	SQASSR**S**SSR(**pS**)RNS	NT	NT	NT	NT	100	NT	111	312	418
SCV2-N [180-193]	SQASSR**S**SSR**S**RNS	NT	NT	NT	NT	101	NT	102	123	1278
SCV2-N [187-200] - pT198	SSR**S**RNS**S**RNS(**pT**)PG	101	250	152	NT	102	100	1042	157	553
SCV2-N [187-200]	SSR**S**RNS**S**RNS**T**PG	100	145	100	NT	103	100	108	485	1080
SCV2-N [195-209] - pS206	RNS**T**PGS**S**RGT(**pS**)PAR	100	NT	105	105	NT	100	1068	205	NT
SCV2-N [195-209]	RNS**T**PGS**S**RGTSPAR	104	NT	101	104	NT	102	137	151	NT
**Nucleocapsid protein**^**a**^	**Full length**	**100**	**99**	**NT**	**102**	**103**	**100**	**101**	**110**	**101**
Autophosphorylation signal (RLU)		5335	558	514	6175	2772	5636	3035	136	2459

Predicted GSK3 phosphorylation sites are bolded and underlined

*NT* not tested

^a^Relative phosphorylation is shown in comparison to the signal recorded as Relative Light Units (RLU) with each protein kinase incubated in the absence of added peptide substrate with ATP alone (set to 100). Values are averages from duplicate measurements

**Table 2:** In vitro phosphorylation of synthetic peptides modeled after SARS-CoV-2 nucleocapsid protein phosphorylation sites by recombinant protein kinase C-alpha and glycogen synthase kinase-beta using the ADP-Glo method

Peptide	Peptide Sequence	PKCa	GSK3b	PKCa+GSK3b
SCV2-N [177-190] - pS188	RGG**S**QAS**S**RSS(**pS**)RS	3861 ± 71	24,585 ± 645	NT
SCV2-N [177-190]	RGG**S**QAS**S**RSS**S**RS	13,714 ± 502	2161 ± 63	3572 ± 22
SCV2-N [180-193] - pS190	SQASSR**S**SSR(**pS**)RNS	4161 ± 66	2957 ± 3	NT
SCV2-N [180-193]	SQASSR**S**SSR**S**RNS	16,366 ± 261	2407 ± 23	3836 ± 41
SCV2-N [187-200] - pT198	SSR**S**RNS**S**RNS(**pT**)PG	7032 ± 319	23,031 ± 486	NT
SCV2-N [187-200]	SSR**S**RNS**S**RNS**T**PG	12,687 ± 486	2573 ± 49	3692 ± 61
SCV2-N [195-209] - pS206	RNS**T**PGS**S**RGT(**pS**)PAR	1531 ± 47	24,082 ± 381	NT
SCV2-N [195-209]	RNS**T**PGS**S**RGTSPAR	3301 ± 114	3291 ± 57	4262 ± 27
**Nucleocapsid protein**^**a**^	**Full length**	**1032 ± 9**	**2052 ± 90**	**2754 ± 66**
No peptide substrate	Autophosphorylation	1282 ± 51	1995 ± 133	2897 ± 38

Predicted GSK3 phosphorylation sites are bolded and underlined. Results are presented as Mean ± Standard deviation, *n* = 3

*NT* not tested

^a^Relative phosphorylation is shown in comparison to the signal with the nucleocapsid protein incubated in the absence of added substrate with ATP alone (set to 100)

## Discussion

Despite successful vaccine development, drugs against SARS-CoV-2 are still needed to manage active infections. Building on existing knowledge regarding the involvement of GSK3β in infection progression, we aimed to identify compounds active against this key human kinase as potential *coronaviridae* inhibitors. Screening of a targeted library yielded multiple hits of active compounds with greater than 50% inhibition of viral infection, nearly half of which conferred some inhibitory effect against SARS-CoV-2 and HCoV-229E. Compared to the ~ 3% ‘hit’ rate of non-targeted host-directed therapy screens [23, 24] or ~ 0.1% in non-specific screens [25], our results demonstrate a high benefit-to-investment ratio.

The efficacy of GSK3β inhibition strategy against two different coronavirus strains in these screens demonstrates that this strategy is not limited to SARS-CoV [6] and SARS-CoV-2, and thus may be an appropriate target for future drug development against various *Coronaviridae*. This is further supported by the presence of conserved RS domains as the target regions for GSK3 phosphorylation in N protein sequences across coronaviruses [7]. In light of the ongoing coronavirus transmission and protracted drug development and approval timelines, the urgency of the COVID-19 pandemic necessitates exploration of such broad-spectrum antivirals.

A singular viral marker may be insufficient to accurately reflect viral infection load, particularly if the marker has potential target-marker interaction, as in the case of GSK3β and the N protein. The SARS-CoV-2 nucleocapsid served as a reliable measurement of viral infection, resulting in a robust Z’ score for the screen (Z’ = 0.6). The dsRNA marker showed slightly reduced robustness (Z’ = 0.3, Supplementary Table 1), likely due to the cross-reactivity of the antiviral dsRNA antibody to that of the host cell. We found a strong agreement between nucleocapsid and dsRNA levels during the screen, indicating that despite being a target for post-translational modifications by GSK3, the nucleocapsid can be used as a viral infection marker in this type of assay. Relative screen inhibition readouts were similar (mean difference of 10%) to those of nucleocapsid expression. Reductions of the spike protein levels, and media-released virus, measured using plaque assay quantification, confirmed that the effect of GSK3β inhibition extends to SARS-CoV-2 assembly and maturation.

Given reported screen result inconsistencies of compound activity against SARS-CoV-2 across different cell lines [20], including different cell lines is important for any robust screening campaign. To guard against this, we internally validated our screen through the use of different cell lines and found a consistent T-1686568 inhibitory effect. While some host-targeting inhibitors designed for viral entry surface molecules, such as against ACE2 and TMPRSS2, present higher expression variability between cell lines [26], our observations indicate GSK3β is a conserved pathway critical to SARS-CoV-2 infection in many tissues and thus is a strong candidate target for drug development. The broad applicability of targeting GSK3β is further supported through observed SARS-CoV-2 inhibition in patient-derived colon organoids, which contain multiple infectible cell types [27]. While donor numbers greatly limits sample sizes, testing of multiple compound concentrations allows for a proxy dose-response evaluation of efficacy. Future examination of alveolar organoids and in vivo challenges will be required for fully exploring the translational potential of T-1686568.

Although results stemming from Figs. 1b, d, and 4a seems contradicting with N protein levels ranging from complete reduction (Fig. 1b) to accumulation upon treatment (Fig. 3a), it is merely a reflection of differences in experimental design and probes used in the assays. HCS (Fig. 1b) detects nucleocapsid signal associated with virions intensity adjusting to background cellular levels. In contrast in Western analysis (Fig. 1d) shows higher levels of N protein as a result of analyzing total cells extracts. These results are strengthened by data shown in Fig. 4a where we can detect some residual levels of N protein in treated cells. The same phenomenon cannot be attributed to the S-protein (Fig. 4i), hence our suggestion that the N protein is accumulated upon treatment. The strong N protein signal reflects a large proportion of it being degraded upon infection (Fig. 4a). A much weaker ~ 48 KDa and ~ 46 KDa immunoreactive doublet correspond to the intact phosphorylated nucleocapsid protein and the lower the unphosphorylated version respectively, can also be observed. Both the ~ 46 KDa and ~ 48 KDa versions are lost following treatment with T-1686568, despite the extremely strong detection of the degraded protein.

Mass spectrometry studies have revealed that the N protein’s RS domain at residues Ser-176 to Ser-276 undergoes extensive phosphorylation (Supplementary Fig. 4). Using synthetic peptides modeled after these phosphosites, we have shown they are directly phosphorylated by recombinant GSK3β, provided these peptides are pre-phosphorylated at priming sites. Recombinant PKCα was able to phosphorylate several of the priming sites for GSK3β in vitro, although other kinases may be responsible for this step in vivo. Our findings complement the recent in vivo studies of Liu et al. [7], which showed that GSK3 acted upstream of the N protein to mediate its phosphorylation. Through site-directed mutagenesis, they further determined that the Ser-188 and Ser-206 priming phosphosites for recognition by GSK3 were required for phosphorylation-dependent mobility shifts of the N protein. Additionally, Thr-205 may act a priming site for Ser-201 and Ser-197 phosphorylation, a process potentially controlled by GSK3 activity.

The phosphorylation of the RS domain in SARS-CoV and SARS-CoV-2 has been implicated in the regulation of N protein binding to RNA, multimerization and subcellular location [28–32]. Studies with SARS-CoV-2 indicate that changes in the phosphorylation status of the RS domain induces profound alterations in the association of multiple nucleocapsid proteins with a single viral RNA in a structured oligomer with RNA-protein and protein-protein interactions to switch to one that permits more viral genome processing [33]. Following N protein phosphorylation in the RS domain, the RNA-protein complex is able to recruit the stress granule protein G3BP1 and suppress the G3BP-dependent host immune response [34]. In our study, the blockage of N protein phosphorylation by GSK3 appeared to result in partial accumulation of the N protein, and prevention of spike protein production to allow formation of the virus particles.

Other recent studies [33, 35–40] have demonstrated that the N protein also undergoes liquid-liquid phase separation when mixed with RNA; it is predicted through polymer theory that the same multivalent interactions driving phase separation also enable RNA compaction. Our future studies aim to validate the hypothesis that phosphorylation of the SARS-CoV-2 N protein by GSK3β is required to form a physiological ‘phase separation’ state necessary for N protein-RNA assembly and budding.

Phosphorylation also plays a role in spike protein behaviour [41], potentially affecting expression levels and cellular trafficking. Our results support this relationship as lower levels of the spike protein (Fig. 3) matched T-1686568-induced viral titer reduction. Our collective findings across multiple assays strongly demonstrate GSK3β inhibition may serve as an effective antiviral strategy for early intervention in COVID-19 and other coronavirus infections, and recommend a potent GSK3β inhibitor for further translational studies.

## Materials and methods

### Cell culture

Caco-2 cells (ATCC® HTB-37™), Calu-3 cells (ATCC® HTB-55™) and Vero E6 cells (ATCC® CRL-1586™) were cultivated in accordance with ATCC recommendations. Human hepatoma Huh-7.5.1 cells [42] were obtained from APATH LLC and cultivated in Dulbecco’s modified Eagle medium (DMEM) supplemented with 10% fetal bovine serum (FBS), 0.1 mM nonessential amino acids, 1 mM sodium pyruvate, and 10 mM HEPES. Experiments were performed in Huh-7.5.1 and Vero E6 cells below passage 30, and Caco-2 and Calu-3 cells below passage 6. All cells were expanded in a T75 flask with 5% carbon dioxide at 37 °C. Cell density was kept between 0.25 and 2 million cells/mL. Intestinal biopsy-derived colonoids from healthy donors were donated from the Johns Hopkins Conte Digestive Disease Basic and Translational Research Core Center (NIH NIDDK P30-DK089502) and grown using methods by Staab et al. [43]

### Compounds

Compounds were kindly supplied by Takeda Pharmaceuticals, solubilized in 100% dimethyl sulfoxide (DMSO, Sigma) to a concentration of 10 mM and stored at − 20 °C. Compounds were diluted in cell-specific media prior to treatment and limited to fewer than five freeze-thaw cycles per aliquot.

### Viral infection assays

All SARS-CoV-2 infections were carried out in a Biological Contamination Level 3 (BCL3) facility (UBC FINDER) in accordance with the Public Health Agency of Canada and UBC FINDER regulations. SARS-CoV-2 SB2 was isolated by Dr. Samira Mubareka (Sunnybrook Hospital, ON, Canada) [44] and passaged in Vero E6 cells. For experiments, passage 3 of the virus was used with a viral titer of 1.5 × 10^7^ plaque forming units (PFU)/mL. B.1.617.2 (delta) and BA.1 (omicron) variants obtained from BEI Resources (ATCC) and passaged in Vero E6 cells. HCoV-229E was kindly obtained from Dr. Eric Jan, (UBC, BC, Canada) and infections were caried out in a Biological Contamination Level 2+ laboratory in Huh-7.5.1 cells at 33 °C. Cells were seeded at a concentration of 10,000 cells/well in 96-well plates, 24 h prior to infection. To ensure reliable signal-to-noise ratios specific to each infection system, SARS-CoV-2 stocks were diluted in cell-specific media to a multiplicity of infection (MOI) of 1 for Caco-2, Vero E6, and Huh-7.5.1, and MOI of 2 for Calu-3 cells and colonoids. Cells were pre-treated with compounds for 3 h, incubated with the virus for 2 days (except for colonoids, which incubated for 3 days), and followed by fixation of the cells with 3.7% Formalin for 30 mins to inactivate the virus. The fixative was removed and cells washed with PBS, followed by immunostaining of the cells with the mouse primary antibody J2 (dsRNA; Jena Biosciences) and rabbit primary antibody HL344 (SARS-CoV-2 nucleocapsid; GeneTex) at working dilutions of 1:1000 for 1 h at room temperature. Secondary antibodies were used at a 1:2000 dilution and included the goat anti-mouse IgG Alexa Fluor 488 (cat #A11001) and goat anti-rabbit IgG Alexa Fluor 555 (A21428) from Invitrogen with the nuclear stain Hoechst 33342 at 1 μg/mL for 1 h in room temperature in the dark. After antibody removal, plates were covered in aluminum foil until scanning to avoid photobleaching.

### High content screening methodology and parameters

Total number of cells (via nuclei staining) and number of virus-infected cells (via dsRNA and nucleocapsid staining) was measured using the CellInsight CX5 High Content Screening platform (Thermo Fisher), as previously described [24]. Briefly, cell nuclei are stained with Hoechst (33342) for detection at the 350/461 nm wavelength and a region of interest (ROI) marks each cell, which is verified using cell membrane imaging in the bright field. The ROI includes the areas where dsRNA (485/521 nm wavelength) and SARS-CoV-2 nucleocapsid (533/588 nm wavelength) are localized. The software (HCS Studio Cell Analysis Software, version 4.0) tallies ROIs where dsRNA and nucleocapsid are located within stained nuclei by compiling pixel area and intensity measurements. The fluorescence emitted by the dsRNA and nucleocapsid are then quantified for each well. The total intensity of each well indicates the viral load and is normalized against non-infected cells and untreated infected cells. Cell loss (from cytotoxicity or poor adherence) was quantified using the same stain (Hoechst 33342) and was used to normalize the changes in viral load resulting from a decrease in cell numbers. Based on average variation in control treatments between experiments, significant cell loss was considered a loss of > 20% cells relative to the non-infected control.

### Confocal microscopy

Huh 7.5.1 cells were seeded at 100,000/slide in a 24-well plate and cultured in DMEM+ 10% FBS for 24 h. They were subsequently infected with SARS CoV-2 at MOI of 2 for 1 h in a 5% carbon dioxide incubator at 37 °C. The inoculum removed and replaced with complete media containing 1% DMSO or 10 μM GSK3β inhibitor. Uninfected cells received only complete media. Cells were left to grow for another 2 days, washed with cold PBS and fixed with 4% paraformaldehyde for 30 mins. The fixative was removed, cells washed with PBS, and permeabilized with 0.1% Triton × 100 for 4 min. Cells were again washed with PBS and blocked with 1% BSA for 1 h. BSA was removed and cells were incubated with the primary antibodies mouse monoclonal antibody to S1 protein (GTX635708, 1:500) and rabbit polyclonal antibody to nucleocapsid protein (GTX635679, 1:1000) for 1 h at room temperature. Secondary antibodies were used at a 1:1000 dilution, incubated for 1 h with Fluoroshield DAPI (Abcam, Ab 104,139) for nuclear staining, removed, washed with PBS, and mounted in aqueous mounting medium. Images were captured on the Zeiss Axio Observer Z1 inverted fluorescent microscope (Zeiss, Germany). The fluorescent intensity was calculated using ImageJ software.

### Dose-response analysis

Intracellular dose-response of viral infection (dsRNA or nucleocapsid signal) in the presence of compounds was performed at dilution factors of 1:1 with three technical replicates in each experiment, and at least three biological replicates per compound concentration. Viral infection levels were interpolated to the negative control (0.1% DMSO, no infection) = 0 and the positive control (0.1% DMSO, with infection) = 100. The GraphPad Prism 9™ (GraphPad Software, Inc.) non-linear regression fit modeling variable slope was used to generate a dose-response curve (Y=Bottom + (Top-Bottom)/(1 + 10^((LogIC50-X)*HillSlope)), constrained to top = 100, bottom = 0.

### Sources of recombinant proteins, peptides and antibodies

The following recombinant SARS-CoV-2 proteins were expressed in *E. coli* and procured from the MRC Protein Phosphorylation and Ubiquitination Unit Reagents and Services at the University of Dundee (Dundee, Scotland): myelin basic protein-spike receptor binding domain (MBP-Spike RBD) amino acids (aa) 319-541 (DU 67753), glutathione S-transferase-nonstructural protein-1 (GST-NSP1) aa 1-180 (DU 66413), GST-NSP2 aa 1-638 (DU 66414); NSP13 aa 1-601 (DU 66417), GST-NSP14 aa 1-527 (DU 66418), GST-NSP15 aa 1-527 (DU 66419), GST-membrane protein aa 1-222 (DU 67699), and GST-nucleocapsid protein aa 1-419 (DU 67726). Additional recombinant SARS-CoV-2 proteins were produced as described [33]. This included fusion proteins with a carrier protein module, a thermophilic family 9 carbohydrate-binding module 208 (CBM9).

All of the protein kinases used in this study were active preparations of recombinant, GST-fusion human proteins expressed in *E. coli* or Sf9 insect cells and sourced from SignalChem (Richmond, BC, Canada). The following kinases were tested: cyclin-dependent kinase 2/cyclin A2 (CDK2/A2; C29-10G), casein kinase 1-alpha-1 (CK1α1; C64-10G), casein kinase 1-delta (CK1δ1; C65-10G), casein kinase 1-gamma-1 (CK1γ1; C68-11G), casein kinase 2-alpha-1 (CK2α1; C70-10G), extracellularly-regulated kinase-1 (ERK1; M29-10G), glycogen synthase kinase-3-beta (GSK3β; G09-10G), cAMP-dependent protein kinase catalytic subunit-alpha (PKACα; P51-10G), and protein kinase C-alpha (PKCα; P61-18G). Production of all synthetic peptides modeled after the sequences in the SARS-CoV-2 proteins was performed by Lifetein (Somerset, NJ, USA).

Peptide affinity-purified rabbit polyclonal antibodies directed against synthetic peptides were acquired from Kinexus Bioinformatics and based on the following SARS-CoV-2 proteins: nucleocapsid aa 156-170 (NNCOV2N-1), ORF1a aa 735-750 – NSP2 (NNCOV2-1A-2), and spike aa 574-588 (NNCOV2S-10).

### Protein kinase reactions

All protein kinase reactions were performed with the ADP-Glo Kinase Assay from ProMega (Madison, WI, USA). Substrate peptides were assayed at a final concentration of ~ 250 μM with 250 μM ATP for 30 min at 30 °C in a final volume of 25 μL. Recombinant nucleocapsid was similarly assayed at a final concentration of 13.4 ng/μL. The concentrations of recombinant protein kinases used were ~ 2 ng/μL.

### Western blotting

To investigate protein expression levels, infected cells were lysed by scraping in the following lysis buffer: 20 mM 3-(*N*-morpholino) propanesulfonic acid (MOPS), pH 7.2, 5 mM EDTA, 2 mM ethylene glycol tetraacetic acid (EGTA), 0.5% (vol/vol) Triton X-100, 30 mM NaF, 20 mM Na_4_P_2_O_7_, 1 mM Na_3_VO_4_, 40 mM β-glycerophosphate, 1 mM dithiothreitol (DTT), 1 mM phenylmethylsulfonyl fluoride, 3 mM benzamidine, 5 μM pepstatin A, and 10 μM leupeptin and 0.5 μM Aprotonin, 0.5 mM Tris (2-carboxyethyl) phosphine hydrochloride (TCEP)- pH- 9.0. Cells were briefly sonicated and 2-Nitro-5-thiocyanatobenzoic acid (NTCB) was added to a final concentration of 6 mM. Tubes were rotated several times to thoroughly expose the contents to lysis buffer and ensure virus inactivation, and samples were subsequently frozen at − 20 °C. Similarly-treated infected cells were tested for plaque formation using a standard PFU test to confirm the lysis buffer deactivated viral particles prior to removal of the assay tubes from BCL3. The tubes were incubated in a 37 °C water bath for 15 min and then subjected to ultracentrifugation at 100,000×*g* for 30 mins. The resulting samples were resolved by SDS-PAGE and transferred onto a nitrocellulose membrane. The blot was probed with the designated antibodies and developed by enhanced chemiluminescence.

### Statistical analysis and artwork

Statistical analyses were performed and figures were prepared using GraphPad Prism 9™ (GraphPad Software, Inc.). Imbedded blots and fluorescent images were generated using GIMP^©^. Chou-Talalay synergy analysis was performed using CompuSyn (ComboSyn Inc.). CI values < 1 are defined as synergistic, CI = 1 as additive, and CI > 1 as antagonistic.

## Supplementary Information

**Additional file 1: Supplemental Table 1.** Focused GSK3β inhibitors screen effect on HCoV-229E and SARS-CoV-2 infected Huh-7.5.1 cells at 10 μM. **Supplemental Fig. S1.** Relationship between inhibition readout using dsRNA and SARS-CoV-2 nucleocapsid for all screened compounds. S = simple linear regression analysis resulted in a y = 1.067x relationship, with 95%CI of 0.9741 to 1.160. **Supplemental Fig. S2.** GSK3β inhibitor activity with (grey) and without (white) pretreatment of seven active inhibitors. Inhibition measured using a H-CoV-229E model of infection, capturing dsRNA immunofluorescence two days post infection. Inhibition interpolated to mock infection and media control. Error bars = SD from three experiments. **Supplemental Fig. S3.** in vitro inhibition of GSK3. ADP-Glo™ Kinase Assay normalized to the negative control (100% kinase activity), dose-response was analyzed by non-linear regression. **Supplemental Fig. S4.** Synergy experiment. Dose-response combination of Remdesivir (Rem) and T-1686568 (T2) using CompuSyn V1.0. Representative experiment of two independent experiments. **Supplemental Fig. S5.** Reduction of cellular syncytia formation and filopodial protrusions associated with 10 μM T-1686568 treatment. **Supplemental Fig. S6.** Confirmed phosphorylated sites in the SARS-CoV-2 nucleocapsid protein. The frequency of literature references for individual phosphosites is shown as documented on the Phosphosite Plus website (www.phosphosite.org) and numbered according to their position in the SARS-CoV-2 nucleocapsid protein. **Supplemental Fig. S7.** Locations of expected positions of target proteins and peptides are circled in the dot blots in Fig. 4g.

## Data Availability

The datasets generated during and/or analysed during the current study are available from the corresponding author on reasonable request.
