# Supplementary material for: Inhibition of glycogen synthase kinase-3-beta (GSK3β) blocks nucleocapsid phosphorylation and SARS-CoV-2 replication
Source: Mol Biomed. 2022 Dec 12;3:43. doi: 10.1186/s43556-022-00111-1 (PMC9742639; doi:10.1186/s43556-022-00111-1)

Inhibition of glycogen synthase kinase-3-beta (GSK3β) blocks nucleocapsid phosphorylation and SARS-CoV-2 replication

Tirosh Shapira^1,2^, Selvarani Vimalanathan^1^, Celine Rens^1^, Virginia Pichler^2^, Sandra Peña-Díaz^2^, Grace Jordana^2^, William Rees^1^, Dirk F.H. Winkler^3^, Iqbal Sarai^3^, Theodore Steiner^1^, François Jean^2^, Steven Pelech ^1,3^, Yossef Av-Gay^*,1,2^

**Supplemental information.**

**Supplemental Table 1:** Focused GSK3β inhibitors screen effect on HCoV-229E and SARS-CoV-2 infected Huh-7.5.1 cells at 10 µM.

| Z' |  | 0.7 | 0.3 | 0.7 | 0.5 |  |  |
| --- | --- | --- | --- | --- | --- | --- | --- |
| **#** | **Takeda ID** | **HCoV-229E inhibition (% dsRNA)** | **SARS-CoV-2 inhibition (% dsRNA)** | **SARS-CoV-2 inhibition (% Nucleocapsid) 1** | **SARS-CoV-2 inhibition (% Nucleocapsid) 2** | **Average SARS-CoV-2 Nucleocapsid inhibition** | **Average cell loss (%)** |
| 1 | P-0181532 | 0.0 | 47.6 | 50.0 | 26.4 | 38.2 | 15.5 |
| 2 | P-0388342 | 0.0 | 23.1 | 21.7 | 8.1 | 14.9 | 20.7 |
| 3 | P-0717632 | 97.6 | 68.2 | 75.2 | 83.3 | 79.3 | 24.4 |
| 4 | P-0766122 | 0.0 | 30.3 | 11.7 | 20.0 | 15.8 | 34.1 |
| 5 | P-0780332 | 0.0 | 0.6 | 0.0 | 0.0 | 0.0 | 9.2 |
| 6 | P-0817042 | 0.0 | 0.0 | 0.0 | 31.3 | 15.7 | 0.0 |
| 7 | P-0861532 | 0.0 | 36.5 | 18.5 | 40.5 | 29.5 | 25.0 |
| 8 | P-0866122 | 0.0 | 48.9 | 46.7 | 17.2 | 31.9 | 32.1 |
| 9 | P-0993232 | 0.0 | 0.0 | 0.0 | 2.1 | 1.0 | 13.6 |
| 10 | P-1071942 | 43.9 | 0.0 | 0.0 | 0.0 | 0.0 | 15.7 |
| 11 | P-1112942 | 10.8 | 0.0 | 9.5 | 28.5 | 19.0 | 9.2 |
| 12 | P-1299432 | 0.0 | 0.0 | 0.0 | 21.1 | 10.6 | 9.9 |
| 13 | P-1312942 | 0.0 | 0.0 | 0.0 | 0.0 | 0.0 | 22.5 |
| 14 | P-1517932 | 0.0 | 26.2 | 13.9 | 37.8 | 25.8 | 16.7 |
| 15 | P-1756242 | 0.0 | 20.1 | 0.0 | 0.0 | 0.0 | 13.9 |
| 16 | P-1766122 | 77.0 | 0.0 | 0.0 | 3.4 | 1.7 | 27.7 |
| 17 | P-1996542 | 0.0 | 0.0 | 0.0 | 0.0 | 0.0 | 0.0 |
| 18 | P-2180632 | 75.9 | 19.0 | 9.2 | 0.0 | 4.6 | 33.0 |
| 19 | P-2253942 | 98.0 | 68.9 | 47.8 | 36.7 | 42.2 | 25.3 |
| 20 | P-2405532 | 0.0 | 0.0 | 0.0 | 0.0 | 0.0 | 5.8 |
| 21 | P-2432522 | 96.1 | 36.4 | 35.8 | 57.3 | 46.5 | 21.0 |
| 22 | P-2517632 | 0.0 | 23.4 | 4.1 | 0.0 | 2.1 | 3.7 |
| 23 | P-2719981 | 0.0 | 0.0 | 0.0 | 0.0 | 0.0 | 64.3 |
| 24 | P-2792732 | 0.0 | 5.1 | 0.0 | 8.5 | 4.2 | 17.7 |
| 25 | P-2817632 | 14.9 | 21.1 | 0.0 | 32.3 | 16.1 | 8.5 |
| 26 | P-2889432 | 0.0 | 79.5 | 61.2 | 46.6 | 53.9 | 20.3 |
| 27 | P-2966122 | 0.0 | 19.1 | 0.6 | 43.2 | 21.9 | 23.0 |
| 28 | P-3517632 | 0.0 | 0.0 | 5.2 | 27.7 | 16.5 | 10.9 |
| 29 | P-3772732 | 30.7 | 13.1 | 0.0 | 8.3 | 4.1 | 36.0 |
| 30 | P-3817632 | 87.7 | 100.0 | 70.8 | 72.0 | 71.4 | 12.7 |
| 31 | P-3866122 | 0.0 | 11.1 | 0.0 | 4.2 | 2.1 | 40.1 |
| 32 | P-3890332 | 0.0 | 7.5 | 0.0 | 0.0 | 0.0 | 9.6 |
| 33 | P-4086132 | 14.1 | 27.4 | 4.4 | 11.2 | 7.8 | 8.4 |
| 34 | P-4179332 | 7.9 | 0.0 | 0.0 | 18.8 | 9.4 | 12.3 |
| 35 | P-4380332 | 85.4 | 23.7 | 0.0 | 48.4 | 24.2 | 13.8 |
| 36 | P-4405532 | 21.2 | 0.0 | 0.0 | 39.9 | 19.9 | 14.8 |
| 37 | P-4423632 | 43.2 | 100.0 | 98.0 | 92.4 | 95.2 | 12.0 |
| 38 | P-4817632 | 0.0 | 43.2 | 1.9 | 0.0 | 1.0 | 8.3 |
| 39 | P-4890332 | 0.0 | 37.5 | 22.4 | 0.0 | 11.2 | 17.8 |
| 40 | P-4993232 | 0.0 | 1.1 | 0.0 | 9.1 | 4.5 | 0.0 |
| 41 | P-5112942 | 0.0 | 13.4 | 19.8 | 19.7 | 19.8 | 32.1 |
| 42 | P-5201042 | 0.0 | 10.4 | 0.0 | 41.4 | 20.7 | 29.0 |
| 43 | P-5266942 | 94.3 | 0.0 | 0.0 | 51.6 | 25.8 | 14.0 |
| 44 | P-5283532 | 0.0 | 16.3 | 0.0 | 0.0 | 0.0 | 0.0 |
| 45 | P-5536732 | 0.0 | 35.7 | 23.5 | 12.4 | 18.0 | 4.0 |
| 46 | P-5603902 | 0.0 | 0.0 | 0.0 | 15.9 | 8.0 | 60.8 |
| 47 | P-5694332 | 0.8 | 0.0 | 0.0 | 12.2 | 6.1 | 8.8 |
| 48 | P-5704332 | 95.6 | 26.7 | 17.8 | 54.3 | 36.0 | 8.2 |
| 49 | P-5782442 | 60.0 | 100.0 | 93.3 | 94.4 | 93.8 | 16.1 |
| 50 | P-5817632 | 39.3 | 24.4 | 0.0 | 47.9 | 24.0 | 2.4 |
| 51 | P-5866122 | 0.0 | 12.6 | 0.0 | 14.8 | 7.4 | 34.4 |
| 52 | P-5868332 | 12.3 | 8.0 | 12.6 | 1.1 | 6.9 | 14.9 |
| 53 | P-5908342 | 91.5 | 60.5 | 76.0 | 48.4 | 62.2 | 21.7 |
| 54 | P-6378522 | 76.2 | 18.5 | 21.1 | 43.8 | 32.5 | 11.3 |
| 55 | P-6579432 | 36.2 | 11.6 | 0.0 | 27.2 | 13.6 | 10.5 |
| 56 | P-6600632 | 0.0 | 12.6 | 20.5 | 14.0 | 17.2 | 12.3 |
| 57 | P-6694332 | 0.0 | 21.6 | 0.0 | 13.0 | 6.5 | 12.1 |
| 58 | P-6849132 | 91.1 | 35.2 | 7.5 | 50.8 | 29.1 | 23.9 |
| 59 | P-6866122 | 0.0 | 44.5 | 27.9 | 28.4 | 28.2 | 41.8 |
| 60 | P-6868332 | 0.0 | 0.0 | 0.0 | 0.0 | 0.0 | 18.7 |
| 61 | P-7079522 | 17.5 | 0.0 | 8.1 | 24.5 | 16.3 | 19.7 |
| 62 | P-7284532 | 36.8 | 0.0 | 0.0 | 0.0 | 0.0 | 7.5 |
| 63 | P-7657632 | 100.0 | 100.0 | 100.0 | 86.2 | 93.1 | 25.4 |
| 64 | P-7694332 | 0.0 | 0.0 | 0.0 | 0.0 | 0.0 | 9.1 |
| 65 | P-7833142 | 2.9 | 0.0 | 0.0 | 28.6 | 14.3 | 31.9 |
| 66 | P-7933732 | 46.2 | 20.3 | 6.7 | 1.7 | 4.2 | 9.2 |
| 67 | P-8060932 | 0.0 | 12.1 | 0.0 | 20.4 | 10.2 | 26.7 |
| 68 | P-8405242 | 95.7 | 5.5 | 0.0 | 2.9 | 1.4 | 24.9 |
| 69 | P-8431332 | 0.0 | 0.0 | 14.2 | 0.0 | 7.1 | 14.5 |
| 70 | P-8536732 | 37.6 | 0.0 | 0.7 | 7.1 | 3.9 | 13.6 |
| 71 | P-8602232 | 0.0 | 0.0 | 0.0 | 5.0 | 2.5 | 27.1 |
| 72 | P-8733142 | 0.0 | 16.0 | 0.0 | 0.0 | 0.0 | 45.6 |
| 73 | P-8866122 | 0.0 | 16.6 | 0.0 | 49.8 | 24.9 | 33.9 |
| 74 | P-8890332 | 33.7 | 0.0 | 12.8 | 23.3 | 18.0 | 17.1 |
| 75 | P-9071942 | 94.2 | 100.0 | 100.0 | 89.1 | 94.5 | 29.9 |
| 76 | P-9101042 | 68.3 | 25.1 | 34.7 | 13.5 | 24.1 | 7.8 |
| 77 | P-9227932 | 98.2 | 54.1 | 47.3 | 33.1 | 40.2 | 16.6 |
| 78 | P-9332522 | 76.1 | 0.0 | 0.0 | 42.2 | 21.1 | 28.0 |
| 79 | P-9431332 | 0.0 | 7.8 | 8.2 | 0.0 | 4.1 | 5.5 |
| 80 | P-9657632 | 0.0 | 53.7 | 23.0 | 43.6 | 33.3 | 33.9 |
| 81 | P-9817632 | 41.5 | 0.0 | 0.0 | 8.6 | 4.3 | 17.8 |
| 82 | P-9866122 | 97.3 | 40.8 | 0.0 | 43.7 | 21.9 | 12.4 |
| 83 | P-9958042 | 0.0 | 0.0 | 2.1 | 14.1 | 8.1 | 15.3 |

**Supplemental Figure S1:** Relationship between inhibition readout using dsRNA and SARS-CoV-2 nucleocapsid for all screened compounds. S = simple linear regression analysis resulted in a y=1.067x relationship, with 95%CI of 0.9741 to 1.160.


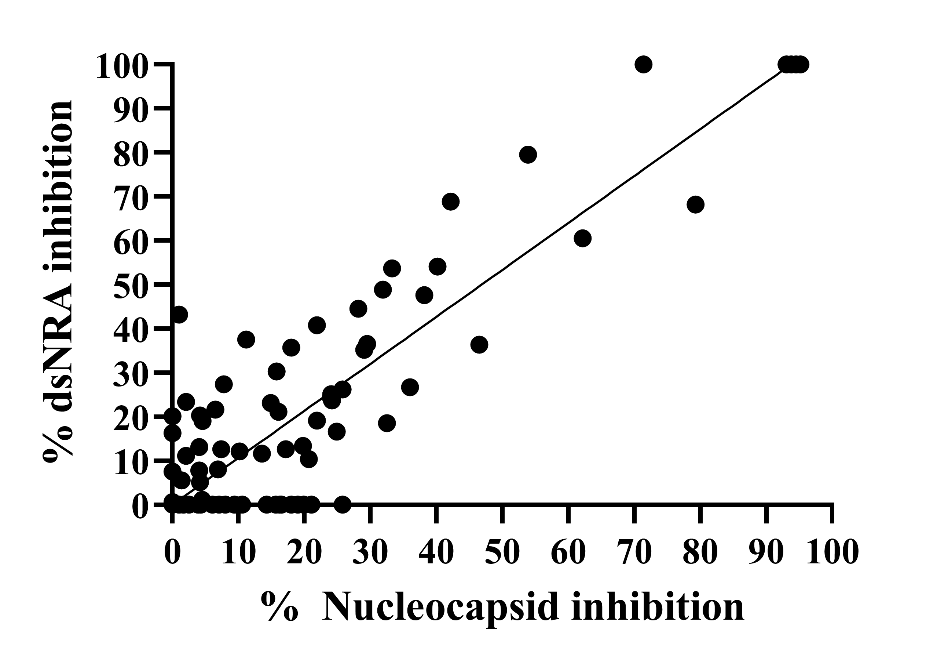


**Supplemental Figure S2:** GSK3β inhibitor activity with (grey) and without (white) pretreatment of seven active inhibitors. Inhibition measured using a H-CoV-229E model of infection, capturing dsRNA immunofluorescence two days post infection. Inhibition interpolated to mock infection and media control. Error bars = SD from three experiments.


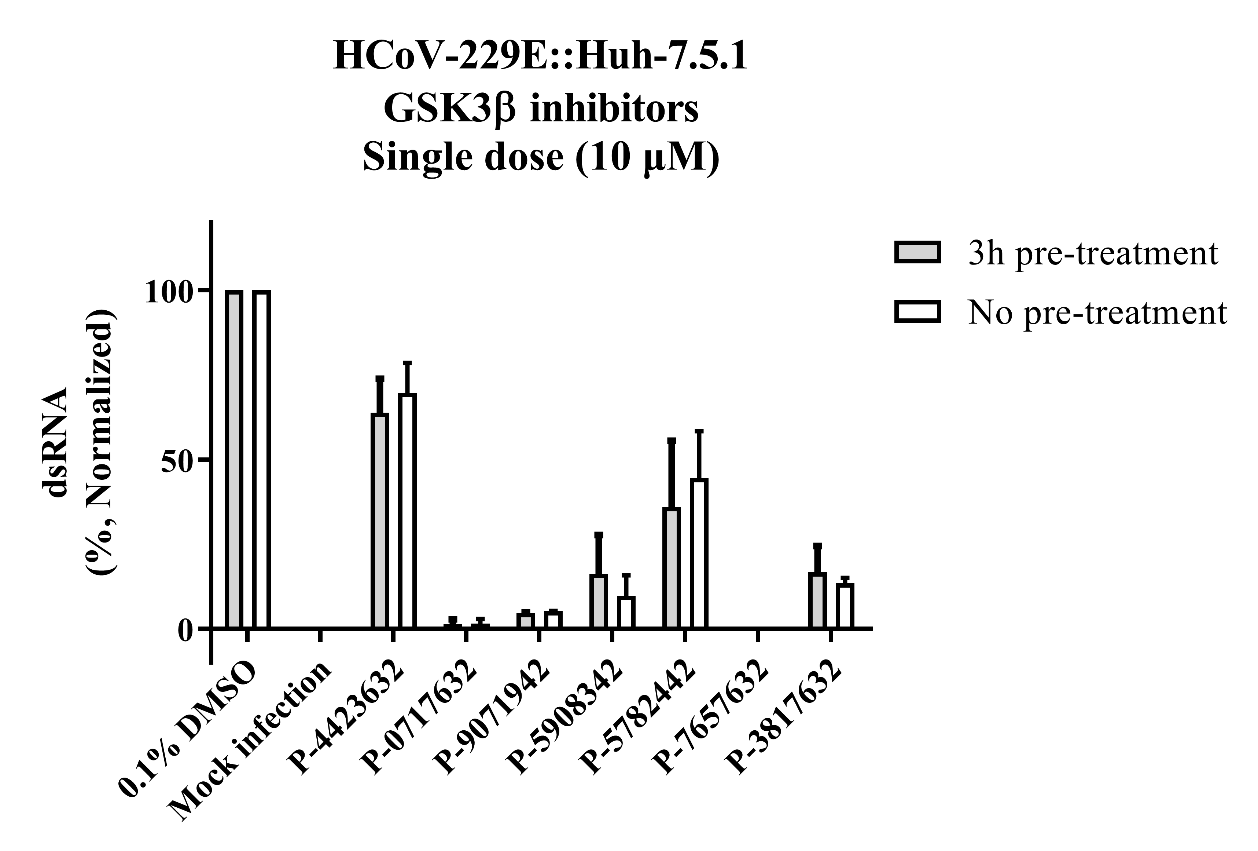


**Supplemental Figure S3:** *in vitro* inhibition of GSK3. ADP-Glo^TM^ Kinase Assay normalized to the negative control (100% kinase activity), dose-response was analyzed by non-linear regression.

**
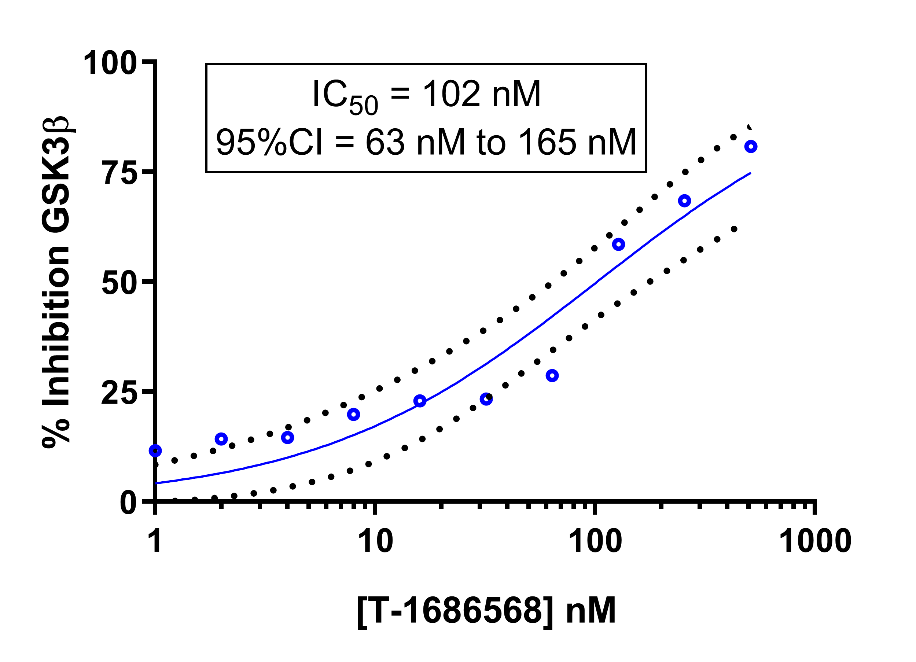
**

**Supplemental Figure S4:** Synergy experiment. Dose-response combination of Remdesivir (Rem) and T-1686568 (T2) using CompuSyn V1.0. Representative experiment of two independent experiments.


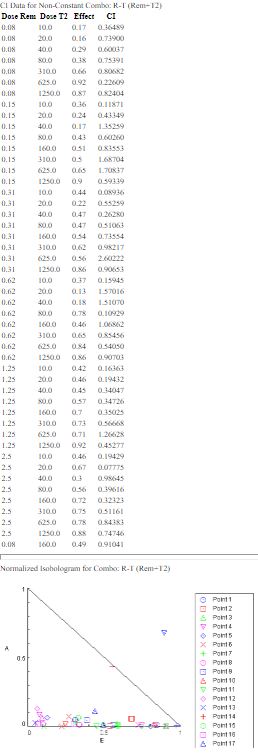


**Supplemental Figure S5:** Reduction of cellular syncytia formation and filopodial protrusions associated with 10 µM T-1686568 treatment

**
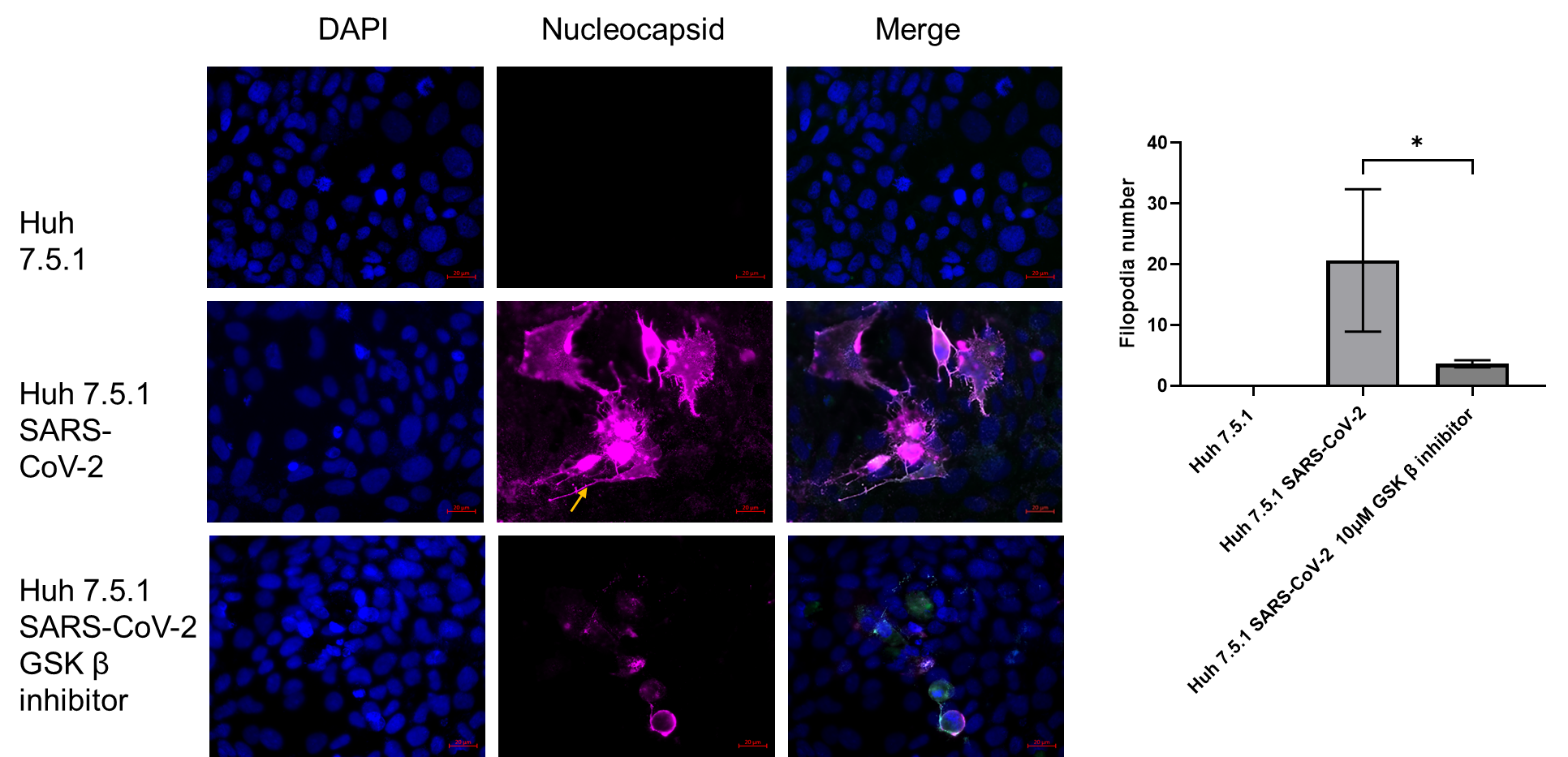
**

**Supplemental Figure S6:** Confirmed phosphorylated sites in the SARS-CoV-2 nucleocapsid protein. The frequency of literature references for individual phosphosites is shown as documented on the Phosphosite Plus website (www.phosphosite.org) and numbered according to their position in the SARS-CoV-2 nucleocapsid protein.


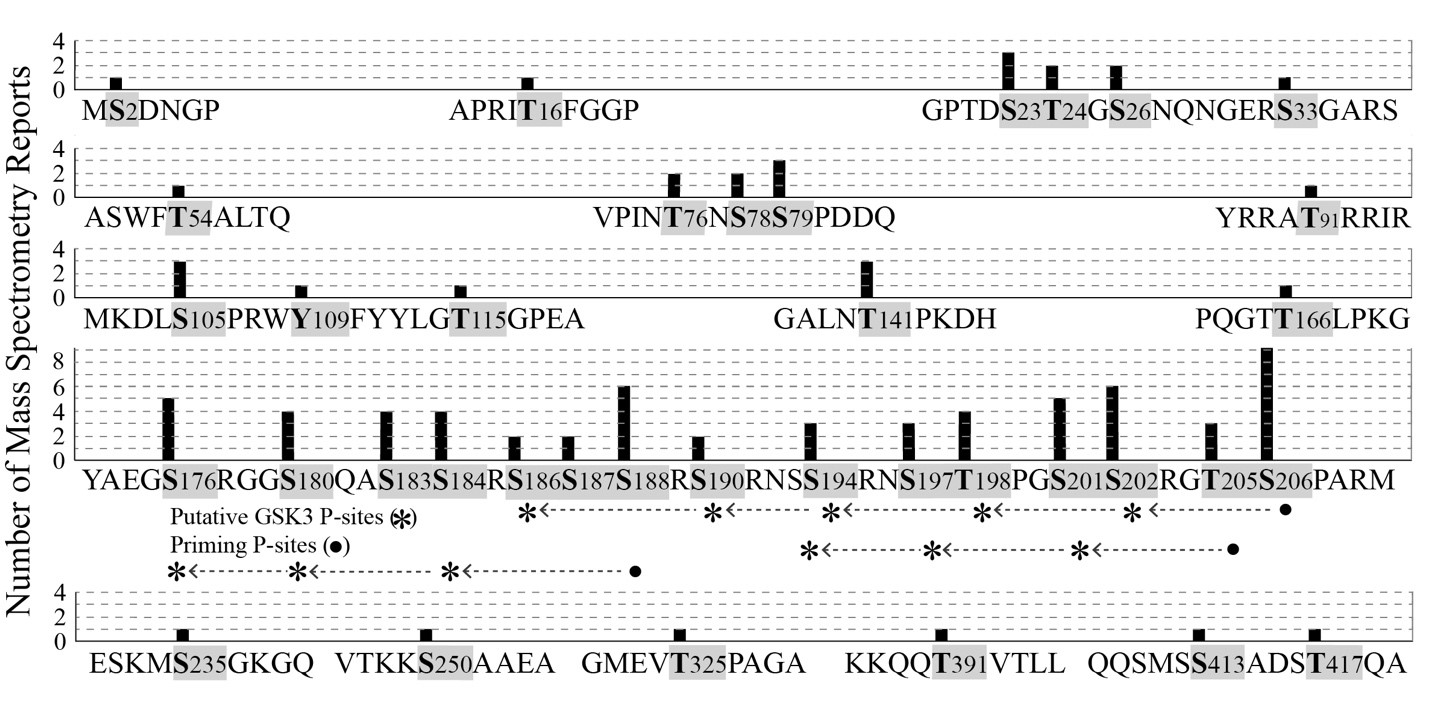


**Supplemental Figure S7:** Locations of expected positions of target proteins and peptides are circled in the dot blots in Fig. 4g


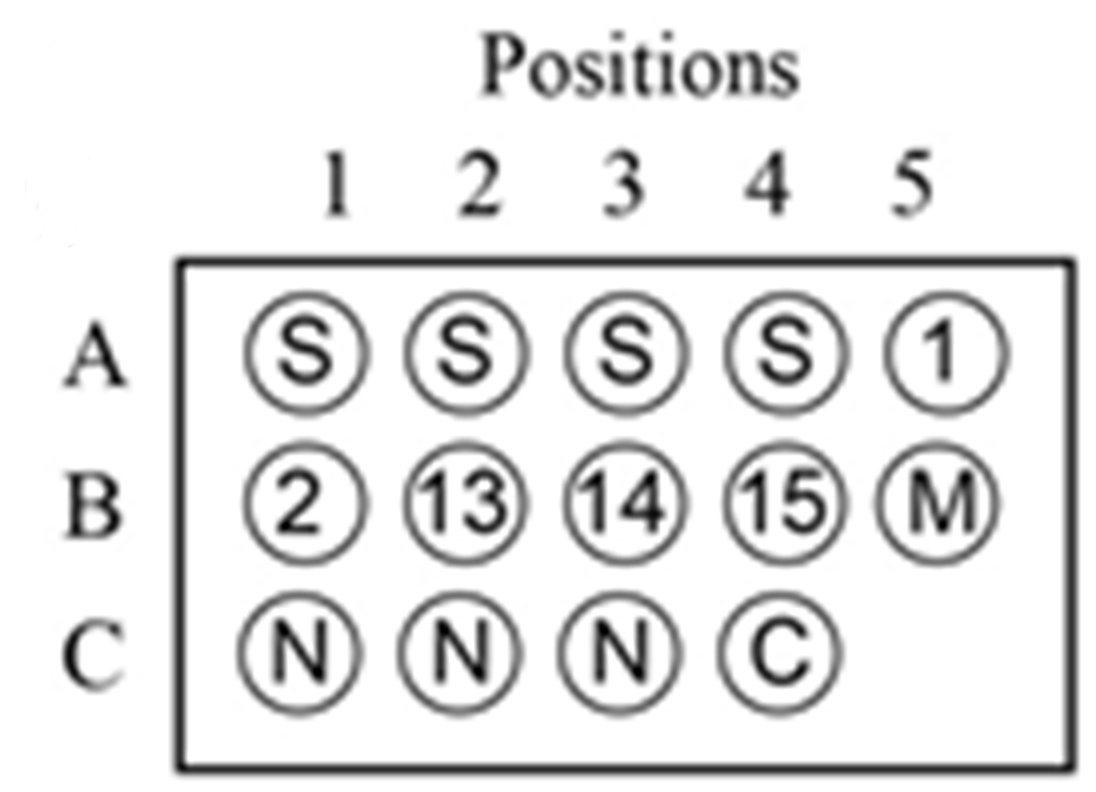


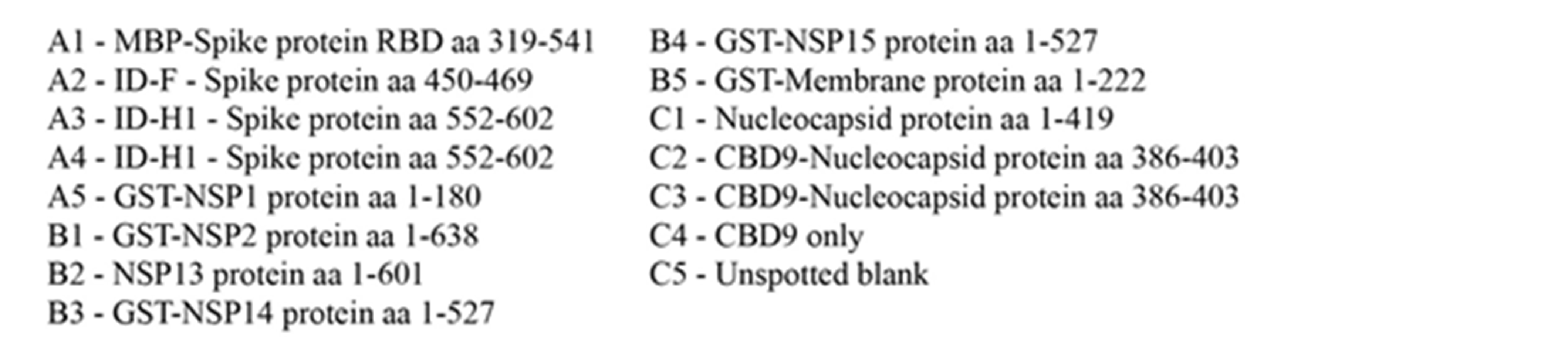

Supplement: Supplementary file 1 — Additional file 1: Supplemental Table 1. Focused GSK3β inhibitors screen effect on HCoV-229E and SARS-CoV-2 infected Huh-7.5.1 cells at 10 μM. Supplemental Fig. S1. Relationship between inhibition readout using dsRNA and SARS-CoV-2 nucleocapsid for all screened compounds. S = simple linear regression analysis resulted in a y = 1.067x relationship, with 95%CI of 0.9741 to 1.160. Supplemental Fig. S2. GSK3β inhibitor activity with (grey) and without (white) pretreatment of seven active inhibitors. Inhibition measured using a H-CoV-229E model of infection, capturing dsRNA immunofluorescence two days post infection. Inhibition interpolated to mock infection and media control. Error bars = SD from three experiments. Supplemental Fig. S3. in vitro inhibition of GSK3. ADP-Glo™ Kinase Assay normalized to the negative control (100% kinase activity), dose-response was analyzed by non-linear regression. Supplemental Fig. S4. Synergy experiment. Dose-response combination of Remdesivir (Rem) and T-1686568 (T2) using CompuSyn V1.0. Representative experiment of two independent experiments. Supplemental Fig. S5. Reduction of cellular syncytia formation and filopodial protrusions associated with 10 μM T-1686568 treatment. Supplemental Fig. S6. Confirmed phosphorylated sites in the SARS-CoV-2 nucleocapsid protein. The frequency of literature references for individual phosphosites is shown as documented on the Phosphosite Plus website (www.phosphosite.org) and numbered according to their position in the SARS-CoV-2 nucleocapsid protein. Supplemental Fig. S7. Locations of expected positions of target proteins and peptides are circled in the dot blots in Fig. 4g. [file 43556_2022_111_MOESM1_ESM.zip › Revised_Supplemental Information_17_11.docx]
